# Factors associated with delays in seeking post abortion care among women in Kenya

**DOI:** 10.1186/s12884-015-0660-7

**Published:** 2015-10-07

**Authors:** Michael M. Mutua, Beatrice W. Maina, Thomas O. Achia, Chimaraoke O. Izugbara

**Affiliations:** African Population and Health Research Center, 2nd Floor APHRC Campus, Manga Close Off Kirawa Road, P.O. Box 10787–00100, Nairobi, Kenya; School of Public Health, University of Witwatersrand, Johannesburg, South Africa

**Keywords:** Post abortion, Care seeking, Delays, Unintended pregnancy, Kenya

## Abstract

**Background:**

Delays in seeking quality post abortion care services remain a major contributor to high levels of mortality and morbidity among women who experience unsafe abortion. However, little is known about the causes of and factors associated with delays in seeking care among women who suffer complications of unsafe abortion. This study looks at factors that are associated with delays in seeking post-abortion care among women in Kenya.

**Methods:**

Data for this study were from a nationally representative sample of 350 healthcare facilities that participated in the 2012 Incidence and Magnitude of Unsafe Abortion study in Kenya. Data included socio-demographic characteristics, reproductive health and clinical histories from all women treated with PAC during a one-month data collection period.

**Results:**

Delay in seeking care was associated with women’s age, education level, contraceptive history, fertility intentions and referral status.

**Conclusions:**

There is need to improve women’s access to quality sexual and reproductive health information and services, contraception and abortion care. Improving current PAC services at lower level facilities will also minimize delays resulting from long referral processes.

## Background

Eighty-six percent of the estimated 43.8 million annual global abortions occur in the developing world [1]. In Africa, the majority (97 %) of these abortions are unsafe [1]; performed either by persons lacking the necessary skills or in an environment lacking the minimal medical standards, or both [2]. In Kenya, unsafe abortion is common and a major contributor to maternal morbidity and mortality [3–6]. The management and treatment of complications of unsafe abortion consume a substantial amount of scarce health systems resources [7]. Clinical data collected from Kenyatta National Hospital in the 80s and 90s show that incomplete abortions accounted for more than half of all the gynecological admissions. Many of these cases required long hospital stays, intensive care, and attendance by highly-skilled, but scarce health providers [8].

In Kenya, abortion is only permitted to save a woman’s life or health [9] and despite the restrictions surrounding it, abortion still remains widespread and often performed clandestinely and unsafely [5, 10]. The bulk of research on unsafe abortion in Kenya has addressed questions related to its incidence [8], risk factors [11] and cost [4]. Little emphasis has been placed on understanding some of the pathways to severity of complications, including care-seeking behavior. Survival from complications arising from unsafe abortion is closely associated with promptness in seeking PAC [12]. Delays in arriving at a health care facility and in being served by a provider [13] can determine the severity of complications of unsafe abortion and can raise a woman’s mortality risk. Several studies have linked these delays to cost of care and distance to the nearest health facility [14, 15]. So far, not many studies have focused on understanding the factors associated with delays in seeking care, which have been clearly demonstrated as a major factor in determining the level of severity of complications after securing an unsafe abortion [12, 16]. This paper utilizes data from a recent study on incidence and complications of unsafe abortion in Kenya to characterize delays in seeking PAC services in Kenya. Findings from this study will demonstrate the need for addressing this specific pathway of complications from unsafe abortion.

## Methods

### Study design

This study is descriptive, analysing cross-sectional data obtained from women seeking PAC in healthcare facilities over a 30-day period in sampled facilities.

### Data and procedures

This paper uses data from the 2012 National Incidence and Magnitude of Unsafe Abortion Study led by the African Population and Health Research Center (APHRC). The study used data from a nationally representative sample of 350 level II-level VI facilities, out of all 2838 PAC-providing facilities in Kenya in 2012. The Kenyan Ministry of Health categorizes the health system into six levels which provide preventive and curative public and private health services as follows; Community health services (Level I); Primary care facilities (Level II and III) comprising of dispensaries, clinics, health centres and maternity homes; county referral health facilities (Levels IV and V) comprising of district/county hospitals, sub-district/county and provincial hospitals; and VI (national referral health facilities comprising of national hospitals) [17]. The study sampled all facilities from level II to Level VI, but excluded dispensaries, which are less likely to offer any PAC services due to staffing and equipment.

### Variables

The primary outcome variable, “delay in seeking care” was based on a set of responses to questions that sought to establish 1) the time it took a woman from the onset of complication (e.g. when first bleeding was spotted) to know that she was experiencing a problem, 2) The time to decide to seek care 3) the time between making the decision to seek care and arrival at health facility. We computed the delay to seek care variable used in this study as the total time of these three different durations as reported by the patient in hours. In addition, we measured the following socio-demographic characteristics: age, level of education, type of residence (rural/urban), and occupation. For all clients including referrals, delay was computed from onset of complications to arrival at health facility at which the patient was observed in this study. For patients who were referred out of the facility of observation, the duration of delay only ends at current facility of observation while patients who were referred to the current facility, the time spent in the referring facility is also captured in the total delay.

### Sample and sampling

As of 31^st^ of January 2012, MoH provided a list of 2838 facilities in levels II to VI with a potential to provide PAC. All level V (17 facilities) and VI (two facilities) facilities were included in the study as well as all (thirty-seven) non-governmental facilities that provide post abortion care or known to provide comprehensive abortion care services. However, for level II-IV facilities, we drew a representative sample using varying sampling fractions at each facility level and region. Therefore, we stratified the total sample according to five geographic regions and five facility levels. We generated these five regions by merging some provinces, which are similar with respect to geographic neighborliness, proximity to shared major healthcare facilities and some level of similarity in selected health-related indicators such as maternal mortality ratios, contraceptive prevalence and total fertility rates. These regions were a) Nairobi and Central b) Nyanza and Western c) Coast and North Eastern d) Rift Valley and e) Eastern provinces. In total 350 facilities were sampled, and a national response rate of 90 % was achieved during data collection. The original survey sample was determined in order to have 80 % power to detect at regional level 10 % difference in the proportion of women with severe complications from unsafe abortion as significant, using a two-sided 5 % significance level.

### Data collection

Trained facility-based healthcare providers who offer PAC services at the sampled facilities collected data from all patients presenting at each of the 326 facilities out of the 350 over a 30-day period. Of the remaining twenty-four facilities, twenty-two did not provide data due to low monthly caseloads while we excluded data from two facilities due to logistical challenges. This gave an average national response rate of 93 % spread according to regions as follows; Nairobi and Central (92 %), Nyanza and Western (99 %), Coast and North Eastern (94 %), Rift Valley (89 %) and Eastern provinces (99 %).

The providers collected patients’ socio-demographic characteristics, reproductive and clinical histories, diagnosis, treatment and clinical procedures performed, post abortion contraception provision, and clinical management outcomes.

### Data management and analysis

We collected all data using paper forms, and later captured into computers using CSPro® and then exported to STATA® 12.1 for consistency checks and analysis. These analyses consisted of descriptive and inferential statistical analysis to describe some of the demand-side characteristics associated with delayed care seeking among women presenting for PAC. The analyses presented in this paper focuses on women who sought PAC. Estimates presented in this paper were weighted using sampling weights generated from the probability of a woman being interviewed in the survey. The sampling fractions were a product of the probability of a facility being selected and accepting to participate and that of a woman participating in the survey based on overall interview response rate at the regional level. To adjust our estimate’s standard errors for design effect due to the complex sampling design above, we generated all statistics presented in this article within STATA’s “svy” platform using the facility level as the primary sampling unit. We summarized delay into median time to care by woman characteristics. For this analysis, given the right-skewedness (positive) of the data (Skeweness = 7.04), we transformed the outcome variable into its natural logarithm, yielding a more symmetric outcome variable (Skeweness = −0.0152). To study factors associated with delayed care seeking, we fitted a random-effects model assuming uniform correlations and estimated the intra-cluster correlation. Past studies have categorized abortion complications into three levels based on clinical signs and symptoms. These classification categories as used in this study were adopted from two main surveys, one in South Africa [18] and another adopted in a study in Kenya [5]. The classifications are outlined in Table 1 below.

**Table 1:** Classification of severity categories of abortion complications

Classification	Signs and symptoms
Severe morbidity	• body temperature of >37.9 ° C
	• organ or system failure
	• generalized peritonitis
	• pulse >119 beats/minute
	• evidence of foreign body or mechanical injury
	• sepsis
	• shock
	• tetanus
	• death
Moderate morbidity	• body temperature between 37.3-37.9 °C
	• adnexal or abdominal tenderness
	• localized peritonitis
	• offensive products of conception
Low morbidity	All other cases

Cases were categorized into the extreme category of abortion complications, and required only one sign or symptom to be counted in that category. (Adopted from Jewkes, Fawcus et al. [28] and Jewkes, Gumede et al. [29]

### Ethical considerations

The study received ethical clearance from the Ethical Review Boards of the Kenya Medical Research Institution (KEMRI), the University of Nairobi/Kenyatta National Hospital, Moi University Teaching and Referral Hospital, and Aga Khan University. The Ministries of Public Health and sanitation and the Medical Services in Kenya and the Institutional Review Board of the Guttmacher Institute also reviewed and approved the study. For ethical considerations, verbal consents were obtained from all women presenting for PAC. Deidentification of records was done before analysis to ensure that data collected on a woman, provider or facility could not be traced back to the source.

## Results

Table 2 shows the socio-demographic and reproductive health characteristics of clients seeking PAC services in Kenya’s healthcare facilities in 2012. It in addition presents the median delays in seeking care by the same characteristics. Over 70 % of the women seeking PAC were aged below 25 years, majority (55 %) of whom were aged between 20 and 24 years. Most women (59 %) reported that their usual residence was rural while 41 % were from urban areas. Sixty-five percent of these PAC patients reported that they were married or living together with a man as if married. On empowerment, specifically educational attainment, 40 % had attained primary level education, while on employment, 42 % were unemployed or housewives with no reported participation in any form of income generation.

**Table 2:** Socio-demographic and reproductive health characteristics of clients seeking post abortion care, Kenya 2012

Socio-demographic characteristics				Reproductive health characteristics			
	%	Median Delay in Hours	N [Un-weighted]		%	Median Delay in Hours	N [Un-weighted]
Age category				Severity level^β^			
10–19 yrs	16.6	72.8	335	Mild	22.8	28.0	723
20–24 yrs	55.0	48.9	1480	Moderate	40.0	30.2	1135
25 + yrs	28.4	49.1	808	Severe	37.2	74.3	773
Residence				Gravidity			
Urban	40.9	50.3	1386	1	27.6	50.5	738
Rural	58.7	49.6	1238	2–3	51.2	49.1	1428
Marital status				4+	21.2	50.7	459
Never married	27.9	58.0	761	Previous abortions			
Married/Living together	64.6	40.7	1700	None	93.2	49.1	2466
Formerly married	7.6	73.6	162	One or more	6.8	98.7	154
Education				Contraception^a^			
No education	9.3	73.9	158	Using modern	29.5	70.1	787
Primary	40.2	49.9	963	Not using	70.5	48.0	1841
Secondary	35.6	50.4	952	Pregnancy wantedness			
Post-secondary	14.8	28.2	543	Wanted then	40.1	27.9	1082
Occupation				Wanted later	27.2	52.6	647
Farmer/unskilled	24.9	53.3	589	Did not want	32.7	71.4	802
Skilled/clerical	20.2	30.1	684	Gestational age^b^			
Student	13.1	52.0	384	First trimester	59.3	50.8	1654
Unemployed/housewife	41.8	49.5	965	Second trimester	40.7	48.1	974
Religion				Referred from			
Catholic	24.4	48.3	606	Not referred	75.2	48.6	1838
Other Christians	64.7	49.5	1767	Referred	24.8	71.0	793
Muslims	8.6	73.5	216				
Others	2.3	97.7	30				
Total	100		2631	Total	100		2631

^a^Defined as the proportion of women who reported using any method of contraception or family planning before the time of conception of current pregnancy and then named a modern form of contraception, with modern contraception defined as pills, injections, implants, male/female sterilization, IUD, male/female condom, diaphragm, foam/jelly, emergency contraception or patch

^b^Based on both client's recall of LMP and doctor's estimation from observation

Table shows weighted proportions and un-weighted counts

Seventy-seven percent of these patients were treated with a moderate or severely complicated abortion. About 7 % of them reported having experienced a previous abortion. Among all PAC patients recorded, 60 % of the pregnancies were unintended, just as high as the 70 % who reported that they were not using a modern contraceptive method at the time of conception. More than half of all women had between 2 and 4 pregnancies (including the current pregnancy which had been terminated). In addition, majority (59 %) of women seeking PAC had first trimester abortions compared to 41 % who presented for PAC while in their 2^nd^ trimester of pregnancy.

We further characterized women seeking care according to delays in seeking care for complications developing after unsafe termination of pregnancies. We used median delay time to further characterize these patients according to the duration in hours that they delayed before seeking care for complications according to socio-economic and demographic characteristics of the women seeking PAC, and the level of complication severity.

Young women aged between 10 and 19 years, with no education, with unwanted pregnancies and those referred from other facilities had longer delays before seeking care. Young women aged less than 20 years reported delays in excess of 3 days before seeking care, almost similar to women who reported having been married before and were either divorced, separated or widowed at the time of seeking care. Similar delays were observed among women with no education and among women confessing Muslim faith as well as un-clarified religious affiliations.

The median delay in hours was highest among women presenting with more severe complications (74 h), while those with moderate complications presented after 30 h. Women with mild complications reported a delayed treatment of about 28 h.

Women with unwanted and mistimed pregnancy sought care much later compared to women with wanted pregnancies (71% and 63 h respectively).

Further investigation shows that while there were longer delays of up to 3 days in seeking care among women referred from other facilities, there were no statistical differences in the level of delay between first trimester and second trimester abortions. Women seeking treatment for 1^st^ trimester abortion complications delayed on average for about 51 h compared to 48 h delay by those seeking treatment for second trimester abortion complications.

Table 2 above shows that women who were referred from other facilities into the facility where they were observed took on average 71 h to reach the referred to facility while those who were treated at the first facility of contact only took about 49 h on average.

We further sought to identify the factors associated with delay in seeking post abortion care.

Table 3 presents the coefficients of influence that predictors have on the natural logarithm of delay in seeking care. The unadjusted model presents the individual coefficients of each of the covariates while the adjusted model presents the coefficients of each covariate controlling for the effect other covariates have on the outcome variable.

**Table 3:** Factors associated with higher natural log of duration before seeking care for complications from unsafe abortion among women in Kenya

		Unadjusted model					Adjusted models				
				95 % Confidence interval					95 % Confidence interval		
		Coef.	P-value	Lower	Upper	Sign	Coef.	P-value	Lower	Upper	Sign
Age category: Ref (10–19 yrs)	20–29 yrs	−0.261	0.008	−0.454	−0.067	^**^	−0.167	0.095	−0.363	0.029	^†^
	30 + yrs	−0.218	0.052	−0.437	0.002	^†^	−0.182	0.157	−0.435	0.070	
Residence: Ref (Rural)	Urban	−0.049	0.534	−0.204	0.106		0.009	0.922	−0.162	0.180	
Region of residence: Ref Central & Nairobi	Coast & N. Eastern	0.175	0.322	−0.171	0.520		0.167	0.374	−0.202	0.537	
	Eastern	0.063	0.728	−0.291	0.416		0.116	0.511	−0.230	0.462	
	Nyanza & Western	−0.028	0.873	−0.378	0.321		−0.043	0.813	−0.398	0.312	
	Rift valley	0.018	0.929	−0.378	0.413		−0.054	0.794	−0.456	0.349	
Marital status: Ref (Never married)	Married/Living together	−0.330	0.000	−0.494	−0.166	^***^	−0.099	0.316	−0.292	0.094	
	Divorced	0.128	0.245	−0.088	0.344		0.168	0.159	−0.066	0.402	
Education: Ref (No education)	Primary	−0.283	0.061	−0.578	0.013	^†^	−0.340	0.046	−0.674	−0.006	^*^
	Secondary	−0.296	0.053	−0.597	0.004	^†^	−0.375	0.042	−0.736	−0.014	^*^
	Post-secondary	−0.356	0.031	−0.679	−0.033	^*^	−0.291	0.165	−0.701	0.120	
Occupation: Ref (Farmer/unskilled)	Skilled/clerical	0.111	0.224	−0.068	0.289		0.055	0.528	−0.116	0.225	
	Student	−0.050	0.608	−0.242	0.142		−0.015	0.882	−0.217	0.186	
	Unemployed/housewife	0.224	0.037	0.013	0.434	^*^	−0.029	0.801	−0.257	0.198	
Religion: Ref (Others)	Catholic	−0.073	0.770	−0.565	0.418		−0.026	0.927	−0.581	0.529	
	Other Christians	−0.092	0.719	−0.591	0.408		0.005	0.987	−0.555	0.564	
	Muslims	0.127	0.615	−0.369	0.623		0.123	0.656	−0.418	0.664	
Severity level^β^: Ref (Mild)	Moderate	0.004	0.964	−0.161	0.169		0.002	0.980	−0.172	0.176	
	Severe	0.712	0.000	0.521	0.903	^***^	0.595	0.000	0.403	0.786	^***^
Gestational age: Ref (First trimester)	Second trimester	0.034	0.636	−0.107	0.176		−0.059	0.450	−0.212	0.094	
Gravidity :Ref (1)	2–3	−0.201	0.018	−0.367	−0.034	^*^	−0.098	0.310	−0.286	0.091	
	4+	0.066	0.510	−0.130	0.262		0.104	0.438	−0.159	0.367	
Previous abortions: Ref (None)	One or more	0.186	0.155	−0.070	0.442		−0.040	0.759	−0.296	0.216	
Contraception: Ref (Not using modern)	Using modern	0.255	0.001	0.111	0.399	^**^	0.165	0.022	0.024	0.305	^*^
Pregnancy wantedness: Ref (Wanted then)	Wanted later	0.417	0.000	0.252	0.582	^***^	0.280	0.001	0.119	0.442	^**^
	Did not want	0.597	0.000	0.411	0.783	^***^	0.323	0.002	0.123	0.523	^**^
Referred: Ref (Not referred)	Referred	0.409	0.000	0.286	0.532	^***^	0.341	0.000	0.206	0.475	^***^

Key: : † p<0.1, * p<0.05, ** p<0.01, *** p<0.001

At individual factor level, bivariate random-effects model shows that delay in seeking care was mainly dependent on woman’s age, marital status, education level, nature of occupation, severity of complication, number of past pregnancies, past contraceptive practice, pregnancy intentions and referral to facility where care was ultimately offered.

All variables, either significant or otherwise in the bivariate level above were included in a multivariable random-effects model. Upon pooling these factors together in moderation, delay in seeking care for abortion complication depended on woman’s age, education level, severity of complication, use of modern contraceptive method, pregnancy intentions and whether or not a patient was referred from another facility. This full model show that a significant amount of variation could be attributed to health facility differences, accounting for about 19 % of the overall variability.

Women aged 20–29 years and those aged 30 years and over were 17 and 18 % (respectively) less likely to have higher natural logs of duration of delays in seeking care compared to adolescents aged 10–19. More educated women reported lower likelihood of longer delays in seeking care, with those with primary education having 34 % lower likelihood, those with secondary education 38 % and those with post-secondary education having 29 % lower likelihood, all compared to those with no education.

On severity of complications, patients with moderate and severe complication had higher likelihood of longer delays, with severe complication having 60 % higher chances of delays. No major differences were observed between mild and moderate complications.

Women who reported use of modern methods of family planning around the time of conception depicted about 17 % higher likelihood of delays. Those with unintended pregnancies were also about 30 % more likely to report higher natural logs of delay. Similar likelihood could be seen among patients referred from other facilities (34 %).

## Discussions

Unsafe abortion remains a major public health challenge in Kenya. In Kenya, majority of abortion services are performed by untrained medical personnel in backstreet clinics without necessary equipment for proper management of patients including performing intrauterine evacuation procedures that may require additional surgical operations [8].

Whenever patients are faced with complications from unsafe abortions, the urgency with which these patients receive treatment makes a difference in the chances of complications developing into severe morbidity or mortality [12, 19].

The factors associated with women’s decisions to seek timely care can be hypothesized at various levels; the women’s level, mainly her efficacy in making such a decision, care provider level based on the provider’s competency and availability of these services, and the policy environment which determines how, where and when these services are delivered and by whom. We measured delay as time between the onset of first signs of complication and the time patients arrived at healthcare facilities, and this transition is often a decision process. First, a patient needs to establish that they are at risk of severe morbidity, and then decide to seek care, and then decide on where to seek care from, and then finally proceed to the place of care [20].

Past studies have established the role of individual characteristics such as, woman’s age, educational attainment and residence, as well as societal factors such as the strength of in-country abortion policies in supporting healthcare needs of women as some of the potential drivers to timely decision making on care seeking [21, 22]. In this study, we found that woman characteristics play key role in determining delay in seeking care for post abortion complications. Other studies have also demonstrated disproportionate stigmatization of healthcare seeking among younger women which would ultimately lead to delay in seeking care [23]. Women’s age influences timing of care seeking [21, 24]. In this study, we have shown that adolescents aged 10–19 were disproportionately exposed to longer delays seek care. Notably, this age group also presented with the highest abortion complication rate [6]. The decision making process in seeking care necessitates some level of competency on the part of the patient, as well as her social support networks. Education and hence woman’s empowerment and autonomy play a significant role in this process [25]. In this case, we found that less educated women delayed to seek care longer irrespective of their age, residence or fertility intentions. These findings go further to support the concept that education and generally, autonomy of decision making on women’s healthcare enables them to make safe rational decisions when faced with health risks [25].

Furthermore, for countries where abortion is legally restricted, the stigma associated with abortion may prohibit women from seeking those services even when within the law [14, 26]. A study carried out in Gabon in 2009 established that women who had undergone an abortion were subject to a higher mortality risk from long delays in initiating care [12]. The concentration of severe complications among women with delayed care seeking further demonstrates the role of social stigma and legal restrictions of abortion care as key in determining complication outcome among women in Kenya.

Referral from lower level facilities exposes women to longer delays, largely due to reduced efficiency in the patient transfer process compared to women who receive treatment at their facility of first contact. The referral process may itself lead to longer delays before administering final treatment or evacuation to a referral patient due to time-loss between facilities. In this study, we found that post abortion care referral patients were more likely to present at the final treating facility much later than their non-referral counterparts do, hence commencement of treatment at heightened complication level.

Additionally, the Kenya’s healthcare provision guidelines do expect PAC services to be offered at level II facilities. However, these level II facilities are the commonly patient’s first line of contact with care providers especially in rural areas. Effectively, these guidelines restrict the Ministry of Health from training staff and equipping the level II facilities to offer PAC. This means that all women presenting at level II facilities are more likely to be referred to higher-level facilities for management exposing women to longer delays to care delivery.

Finally, pregnancy intention was strongly associated with delayed seeking care. Notably, majority of unwanted pregnancies are due to changing intentions between conception and birth, due to lack of assurance of financial support during and after delivery [10]. This study established that women with unintended pregnancies were at higher risks of experiencing longer delays in seeking care for complications, which exposes them to increased complications hence higher risks of maternal mortality.

### Limitations

Despite the data methods used in this survey providing significant improvement on the common challenge of underreporting, the main data limitation in this study is on its generalizability. Data was collected from women presenting at healthcare facilities, which may represent only a special group of women; those who sought care, disregarding women who did not delay at all or who delayed longest because they never sought care in the first place. In addition, providers collected data from women after completing emergency treatment or evacuation procedures and this might have in effect lowered women’s recall of actual time between onset of complications and arrival at health facility.

## Conclusions

In this study, we have identified the extent of post abortion complications risks resulting from delayed care seeking among PAC women in Kenya. We have further highlighted the need for development of youth-friendly PAC services, and integration of PAC within existing youth services (YFS) in the country. These will, among other benefits, assure adolescents of proper PAC information on service sources, costs and availability in a manner that best communicates to this special age groups. The urgency of addressing this group is that the long-term consequences of delays in seeking care would be severer among adolescents from long-term or permanent disabilities, hence longer years of disability and poor quality of life. On referral, there is an urgent need for equipping lower level facilities (such as level II and III) with both staff and equipment, such as MVA kits and medication to support use of less invasive post abortion procedures such as medical abortion. This will ensure that patients receive quality PAC treatment at their first point of contact with a care provider and reduce referral rates to a bare minimum. This study also highlighted the need for increased awareness of the risks of delayed PAC seeking the risks of second trimester abortions and generally the risks of self-induced abortions. Majority of the women studied here who presented for either first or second trimester PAC had either self-induced abortions, or with the help of a friend or relative or even an untrained healthcare provider or agent. This poses the risks of permanent damage of women’s reproductive system hence high exposure to poor quality of life.

In conclusion, this study findings call for greater efforts and evaluation of reproductive health programs instituted to provide services to women, especially young people and expansion of care delivery to include lower level facilities in Kenya and hence cut down the time before treatment for PAC patients. Delay in seeking PAC services increases the risk of maternal mortality, or severe complications, which may have lifelong effects on young women [5, 19, 27]. There is therefore need for improved and more effective referral system that minimizes unnecessary delays in care delivery, and task-shifting measures geared towards increasing service delivery at lower level facilities to reduce the time to care among women experiencing complications from unsafe abortions.
